# Supplementary material for: Bone Metabolism Effects of Medical Therapy in Advanced Renal Cell Carcinoma
Source: Cancers (Basel). 2023 Jan 15;15(2):529. doi: 10.3390/cancers15020529 (PMC9856493; doi:10.3390/cancers15020529)
Supplement: Supplementary file 1 [file cancers-15-00529-s001.zip › cancers-2102371-supplementary.pdf]

Table S1. List of randomized clinical trials that led to the approval of anticancer agents in RCC patients and the reported adverse events related to bone metabolism.

					Adverse Events							
**Clinical Trial**	**Trial Phase (Year of publication)**	**Histology - Line of treatment**	**Experimental Arm**	**Control Arm**	**High Calcium**	**Low Calcium**	**Low Phosphate**	**Low Magnesium**	**High Magnesium**	**Fractures**	**BMD alterations**	**Osteoporosis**
ARCC [1]	Phase III (2007)	ccRCC - FL	Temsirolimus - Temsirolimus + Interferon alfa	Interferon alfa	NR	NR	NR	NR	NR	NR	NR	NR
NCT00098657 [2]	Phase III (2007)	ccRCC - FL	Sunitinib	Interferon alfa	NR	NR	36% (AG)	NR	NR	NR	NR	NR
TARGET [3]	Phase III (2007)	ccRCC - SL	Sorafenib	Placebo	NR	NR	NR	NR	NR	NR	NR	NR
NCT00410124 [4]	Phase III (2008)	ccRCC - SL	Everolimus	Placebo	NR	17% (AG)	32% (AG)	NR	NR	NR	NR	NR
NCT00334282 [5]	Phase III (2010)	ccRCC - FL	Pazopanib	Placebo	NR	33% (AG)	34% (AG)	11% (AG)	NR	NR	NR	NR
AXIS [6]	Phase III (2011)	ccRCC - SL	Axitinib	Sorafenib	6% (AG)	39% (AG)	13% (AG)	NR	NR	NR	NR	NR
COMPARZ [7]	Phase III (2013)	ccRCC - FL	Pazopanib	Sunitinib	NR	NR	36% (AG)*	23% (AG)*	12% (AG)*	NR	NR	NR
NCT00920816 [8]	Phase III (2013)	ccRCC - FL	Axitinib	Sorafenib	NR	NR	NR	NR	NR	NR	NR	NR
CheckMate 025 [9]	Phase III (2015)	ccRCC - SL	Nivolumab	Everolimus	NR	NR	NR	NR	NR	NR	NR	NR
METEOR [10]	Phase III (2015)	ccRCC - SL	Cabozantinib	Everolimus	NR	NR	10% (AG)	16% (AG)	NR	NR	NR	NR
NCT01136733 [11]	Phase II (2015)	ccRCC - SL	Lenvatinib - Everolimus + Lenvatinib	Everolimus	NR	NR	NR	NR	NR	NR	NR	NR
ASPEN [12]	Phase II (2016)	nccRCC - FL	Sunitinib	Everolimus	NR	NR	NR	5% (G1-2)	NR	NR	NR	NR
CABOSUN [13]	Phase II (2016)	ccRCC - FL	Cabozantinib	Sunitinib	NR	NR	NR	NR	NR	NR	NR	NR
CheckMate 214 [14]	Phase III (2018)	ccRCC - FL	Ipilimumab + Nivolumab	Sunitinib	NR	NR	NR	NR	NR	NR	NR	NR

JAVELINE Renal 101 [15]	Phase III (2019)	ccRCC - FL	Axitinib + Avelumab	Sunitinib	NR	NR	NR	NR	NR	NR	NR	NR
KEYNOTE-426 [16]	Phase III (2019)	ccRCC - FL	Axitinib + Pembrolizumab	Sunitinib	NR	NR	NR	NR	NR	NR	NR	NR
TIVO-3 [17]	Phase III (2019)	ccRCC - SL	Tivozanib	Sorafenib	NR	NR	NR	NR	NR	NR	NR	NR
CheckMate 9ER [18]	Phase III (2021)	ccRCC - FL	Cabozantinib + Nivolumab	Sunitinib	NR	NR	14,4% (AG)	13,8% (AG)	NR	NR	NR	NR
CLEAR [19]	Phase III (2021)	ccRCC - FL	Lenvatinib + Pembrolizumab - Lenvatinib + Everolimus	Sunitinib	NR	1,4% (AG)**	NR	NR	NR	NR	NR	NR
SWOG 1500 [20]	Phase II (2021)	nccRCC - FL	Cabozantinib - Crizotinib - Savolitinib	Sunitinib	NR	10% (AG)	11% (AG)	9% (AG)	NR	NR	NR	NR

Adverse events reported in the Table refer to the "experimental arm".
*Adverse events related to Sunitinib.
**Adverse events related to Lenvatinib_Pembrolizumab

Abbreviations: Any Grade (AG) - Clear Cell Renal Cell Carcinoma (ccRCC) – First Line (FL) – Not Clear Cell Renal Cell Carcinoma (nccRCC) - Not Reported (NR) – Renal Cell Carcinoma (RCC) - Subsequent Line (SL) – Von Hippel Lindau (VHL).

References

1. Hudes, G.; Carducci, M.; Tomczak, P.; Dutcher, J.; Figlin, R.; Kapoor, A.; Staroslawska, E.; Sosman, J.; McDermott, D.; Bodrogi, I., *et al.* Temsirolimus, interferon alfa, or both for advanced renal-cell carcinoma. *The New England journal of medicine* **2007**, *356*, 2271-2281. doi:10.1056/NEJMoa066838
2. Motzer, R.J.; Hutson, T.E.; Tomczak, P.; Michaelson, M.D.; Bukowski, R.M.; Rixe, O.; Oudard, S.; Negrier, S.; Szczylik, C.; Kim, S.T., *et al.* Sunitinib versus interferon alfa in metastatic renal-cell carcinoma. *The New England journal of medicine* **2007**, *356*, 115-124. doi:10.1056/NEJMoa065044
3. Escudier, B.; Eisen, T.; Stadler, W.M.; Szczylik, C.; Oudard, S.; Siebels, M.; Negrier, S.; Chevreau, C.; Solska, E.; Desai, A.A., *et al.* Sorafenib in advanced clear-cell renal-cell carcinoma. *The New England journal of medicine* **2007**, *356*, 125-134. doi:10.1056/NEJMoa060655
4. Motzer, R.J.; Escudier, B.; Oudard, S.; Hutson, T.E.; Porta, C.; Bracarda, S.; Grünwald, V.; Thompson, J.A.; Figlin, R.A.; Hollaender, N., *et al.* Efficacy of everolimus in advanced renal cell carcinoma: A double-blind, randomised, placebo-controlled phase iii trial. *Lancet (London, England)* **2008**, *372*, 449-456. doi:10.1016/s0140-6736(08)61039-9
5. Sternberg, C.N.; Davis, I.D.; Mardiak, J.; Szczylik, C.; Lee, E.; Wagstaff, J.; Barrios, C.H.; Salman, P.; Gladkov, O.A.; Kavina, A., *et al.* Pazopanib in locally advanced or metastatic renal cell carcinoma: Results of a randomized phase iii trial. *Journal of clinical oncology : official journal of the American Society of Clinical Oncology* **2010**, *28*, 1061-1068. doi:10.1200/jco.2009.23.9764
6. Rini, B.I.; Escudier, B.; Tomczak, P.; Kaprin, A.; Szczylik, C.; Hutson, T.E.; Michaelson, M.D.; Gorbunova, V.A.; Gore, M.E.; Rusakov, I.G., *et al.* Comparative effectiveness of axitinib versus sorafenib in advanced renal cell carcinoma (axis): A randomised phase 3 trial. *Lancet (London, England)* **2011**, *378*, 1931-1939. doi:10.1016/s0140-6736(11)61613-9
7. Motzer, R.J.; Hutson, T.E.; Cella, D.; Reeves, J.; Hawkins, R.; Guo, J.; Nathan, P.; Staehler, M.; de Souza, P.; Merchan, J.R., *et al.* Pazopanib versus sunitinib in metastatic renal-cell carcinoma. *The New England journal of medicine* **2013**, *369*, 722-731. doi:10.1056/NEJMoa1303989
8. Hutson, T.E.; Lesovoy, V.; Al-Shukri, S.; Stus, V.P.; Lipatov, O.N.; Bair, A.H.; Rosbrook, B.; Chen, C.; Kim, S.; Vogelzang, N.J. Axitinib versus sorafenib as first-line therapy in patients with metastatic renal-cell carcinoma: A randomised open-label phase 3 trial. *The Lancet. Oncology* **2013**, *14*, 1287-1294. doi:10.1016/s1470-2045(13)70465-0
9. Motzer, R.J.; Escudier, B.; McDermott, D.F.; George, S.; Hammers, H.J.; Srinivas, S.; Tykodi, S.S.; Sosman, J.A.; Procopio, G.; Plimack, E.R., *et al.* Nivolumab versus everolimus in advanced renal-cell carcinoma. *The New England journal of medicine* **2015**, *373*, 1803-1813. doi:10.1056/NEJMoa1510665
10. Choueiri, T.K.; Escudier, B.; Powles, T.; Mainwaring, P.N.; Rini, B.I.; Donskov, F.; Hammers, H.; Hutson, T.E.; Lee, J.L.; Peltola, K., *et al.* Cabozantinib versus everolimus in advanced renal-cell carcinoma. *The New England journal of medicine* **2015**, *373*, 1814-1823. doi:10.1056/NEJMoa1510016
11. Motzer, R.J.; Hutson, T.E.; Glen, H.; Michaelson, M.D.; Molina, A.; Eisen, T.; Jassem, J.; Zolnierek, J.; Maroto, J.P.; Mellado, B., *et al.* Lenvatinib, everolimus, and the combination in patients with metastatic renal cell carcinoma: A randomised, phase 2, open-label, multicentre trial. *The Lancet. Oncology* **2015**, *16*, 1473-1482. doi:10.1016/s1470-2045(15)00290-9
12. Armstrong, A.J.; Halabi, S.; Eisen, T.; Broderick, S.; Stadler, W.M.; Jones, R.J.; Garcia, J.A.; Vaishampayan, U.N.; Picus, J.; Hawkins, R.E., *et al.* Everolimus versus sunitinib for patients with metastatic non-clear cell renal cell carcinoma (aspen): A multicentre, open-label, randomised phase 2 trial. *The Lancet. Oncology* **2016**, *17*, 378-388. doi:10.1016/s1470-2045(15)00515-x
13. Choueiri, T.K.; Halabi, S.; Sanford, B.L.; Hahn, O.; Michaelson, M.D.; Walsh, M.K.; Feldman, D.R.; Olencki, T.; Picus, J.; Small, E.J., *et al.* Cabozantinib versus sunitinib as initial targeted therapy for patients with metastatic renal cell carcinoma of poor or intermediate risk: The alliance a031203 cabosun trial. *Journal of clinical oncology : official journal of the American Society of Clinical Oncology* **2017**, *35*, 591-597. doi:10.1200/jco.2016.70.7398
14. Motzer, R.J.; Tannir, N.M.; McDermott, D.F.; Aren Frontera, O.; Melichar, B.; Choueiri, T.K.; Plimack, E.R.; Barthelemy, P.; Porta, C.; George, S., *et al.* Nivolumab plus ipilimumab versus sunitinib in advanced renal-cell carcinoma. *The New England journal of medicine* **2018**, *378*, 1277-1290. doi:10.1056/NEJMoa1712126
15. Motzer, R.J.; Penkov, K.; Haanen, J.; Rini, B.; Albiges, L.; Campbell, M.T.; Venugopal, B.; Kollmannsberger, C.; Negrier, S.; Uemura, M., *et al.* Avelumab plus axitinib versus sunitinib for advanced renal-cell carcinoma. *The New England journal of medicine* **2019**, *380*, 1103-1115. doi:10.1056/NEJMoa1816047

16. Rini, B.I.; Plimack, E.R.; Stus, V.; Gafanov, R.; Hawkins, R.; Nosov, D.; Pouliot, F.; Alekseev, B.; Soulieres, D.; Melichar, B., *et al.* Pembrolizumab plus axitinib versus sunitinib for advanced renal-cell carcinoma. *The New England journal of medicine* **2019**, *380*, 1116-1127. doi:10.1056/NEJMoa1816714
17. Rini, B.I.; Pal, S.K.; Escudier, B.J.; Atkins, M.B.; Hutson, T.E.; Porta, C.; Verzoni, E.; Needle, M.N.; McDermott, D.F. Tivozanib versus sorafenib in patients with advanced renal cell carcinoma (tivo-3): A phase 3, multicentre, randomised, controlled, open-label study. *The Lancet. Oncology* **2020**, *21*, 95-104. doi:10.1016/s1470-2045(19)30735-1
18. Choueiri, T.K.; Powles, T.; Burotto, M.; Escudier, B.; Bourlon, M.T.; Zurawski, B.; Oyervides Juarez, V.M.; Hsieh, J.J.; Basso, U.; Shah, A.Y., *et al.* Nivolumab plus cabozantinib versus sunitinib for advanced renal-cell carcinoma. *The New England journal of medicine* **2021**, *384*, 829-841. doi:10.1056/NEJMoa2026982
19. Motzer, R.; Alekseev, B.; Rha, S.Y.; Porta, C.; Eto, M.; Powles, T.; Grünwald, V.; Hutson, T.E.; Kopyltsov, E.; Méndez-Vidal, M.J., *et al.* Lenvatinib plus pembrolizumab or everolimus for advanced renal cell carcinoma. *The New England journal of medicine* **2021**, *384*, 1289-1300. doi:10.1056/NEJMoa2035716
20. Pal, S.K.; Tangen, C.; Thompson, I.M., Jr.; Balzer-Haas, N.; George, D.J.; Heng, D.Y.C.; Shuch, B.; Stein, M.; Tretiakova, M.; Humphrey, P., *et al.* A comparison of sunitinib with cabozantinib, crizotinib, and savolitinib for treatment of advanced papillary renal cell carcinoma: A randomised, open-label, phase 2 trial. *Lancet (London, England)* **2021**, *397*, 695-703. doi:10.1016/s0140-6736(21)00152-5
